# Long-Term Outcomes of Radiofrequency Ablation for Treatment of Cystic Warthin Tumors versus Solid Warthin Tumors

**DOI:** 10.3390/ijerph18126640

**Published:** 2021-06-21

**Authors:** Chih-Hung Cha, Sheng-Dean Luo, Pi-Ling Chiang, Wei-Chih Chen, Yu-Cheng Tung, Yan-Ye Su, Wei-Che Lin

**Affiliations:** 1 Department of Otolaryngology, Kaohsiung Chang Gung Memorial Hospital, Chang Gung University College of Medicine, Kaohsiung 833401, Taiwan; d070025@cgmh.org.tw (C.-H.C.); rsd0323@cgmh.org.tw (S.-D.L.); jarva@cgmh.org.tw (W.-C.C.); yanyesu@cgmh.org.tw (Y.-Y.S.); 2 Department of Diagnostic Radiology, Kaohsiung Chang Gung Memorial Hospital, Chang Gung University College of Medicine, Kaohsiung 833401, Taiwan; lovage@cgmh.org.tw (P.-L.C.); yctung66@gmail.com (Y.-C.T.)

**Keywords:** warthin tumor, radiofrequency ablation, ultrasound, parotid gland, parotidectomy

## Abstract

Background: To describe the long-term outcomes of radiofrequency ablation (RFA) of parotid Warthin tumors that have different consistencies and locations. Methods: We reviewed ten patients with Warthin tumors undergoing RFA treatment from 2016 to 2019. The mean follow-up was 24.3 ± 13.1 months (range 7–42 months). Results: RFA was performed on 11 tumors in ten patients. Cystic tumors (*n* = 4) had better volume reduction ratios (VRR) than solid tumors (*n* = 7) at month one and month six, following RFA (77.9% vs. 47.3%, 95.1% vs. 80.6%, respectively, *p* = 0.003). Tumors in both superficial lobes and deep lobes (*n* = 7) were larger than tumors in superficial lobes alone (*n* = 4), though there was no difference in VRR after treatment. All residual tumors were found in superficial lobes. There was no increase in residual tumor size. Every patient showed marked cosmetic improvements, with visible tumors becoming non-palpable masses. Conclusions: RFA is a safe and effective treatment for Warthin tumors, with better volume reduction in cystic tumors. Results remained satisfying over the long-term for all residual tumors found in superficial lobes, making it easier for re-intervention if necessary.

## 1. Introduction

Warthin tumors, once known as cyst adenolymphoma, are the second most common benign tumors of the salivary gland, accounting for 15% of all parotid epithelial tumors, following pleomorphic adenoma (60%) [1]. Very few Warthin tumors become malignant (<1%) [2]. They are almost exclusively parotid tumors, though two percent have been found to be multifocal, either synchronous or metachronous, arising within adjacent lymphoid tissue. Around ten percent of these tumors are bilateral and only two percent recur [3]. They most often develop in the lower pole of parotid gland, known as the parotid tail [4].

Although the causes of these tumors remain unknown, most patients, especially those with bilateral involvement, have been cigarette smokers, with a peak incidence in the seventh decade of life [5]. There is a strong male predominance in the prevalence of these tumors, with the male to female ratios ranging from 10:1 to 3:1 [6].

There is some controversy regarding the best approach to treating Warthin tumors. Currently, the most common approach is surgical resection, either superficial parotidectomy, total parotidectomy, or enucleation. Superficial parotidectomy is the most-widely used approach. Even when an experienced surgeon makes use of facial nerve monitoring systems, there is high risk of permanent facial nerve damage associated with parotid surgery. The reported incidences of temporary facial palsy in benign parotid surgery are 60%, 26%, 18%, and 11% for total parotidectomy, superficial parotidectomy, partial superficial parotidectomy, and extracapsular dissection, respectively [7]. Damage may be permanent in 2–3% of all cases [8]. Other complications include postoperative infection, salivary fistula, hematoma/hemorrhage, sialocele, and Frey’s syndrome. Consequently, some surgeons prefer to treat these tumors with more conservative resections, such as enucleation and local excision, though there are some concerns about possible recurrence [7].

Warthin tumors can also be managed conservatively, since their growth rate and risk of malignancy is low [9]. They do grow, however, and are known to double in volume in the space of around nine years, with a growth rate of 8% per year [10]. The larger the tumor, the greater the risk of surgery-related facial nerve injury, so it may not be advisable to delay management. A delay in treatment may allow tumor mass to cause cosmetic problems such as an asymmetric face or a feeling of compression, as well as an increased risk of sialadenitis, a rare infection of the salivary glands [11,12,13].

Previously, we used radiofrequency ablation (RFA) to treat seven Warthin tumors [14]. We found that the ultrasound-guided RFA moving shot technique was a safe and effective alternative treatment for parotid tail Warthin tumors in the seven patients for whom surgical resection was contraindicated, or for those were unwilling to receive surgical resection. However, the follow-up period in that study was short, at only six months. In this study, we revisited this patient group and re-evaluated the efficacy and safety of RFA treatment of Warthin tumors in a slightly larger series of tumors that had different consistencies and locations. We also followed these patients for a longer period.

## 2. Materials and Methods

### 2.1. Patient Population and Evaluation

In this retrospective, observational consecutive case series, we reviewed the treatment of ten patients who received ultrasound-guided percutaneous RFA for the treatment of Warthin tumor from September 2016 to August 2019, at our 2000-bed tertiary referral medical center in Taiwan. The protocol for this study was approved by the institutional review board at our medical center, and informed written consent was signed by all subjects after the procedure was explained in detail. All patients received information about treatment options available for their parotid tumors, including conservative observation, surgery, and RFA.

We included patients who had a pathology-proved Warthin tumor based on ultrasound-guided core needle biopsies, whose tumors exceeded 1 cm in maximum diameter, who reported pressure discomfort or cosmetic complaints, and who preferred RFA treatment. We excluded any patients who had previously received parotid surgery or who concurrently had other lesions of the head and neck. In total, ten male patients were included, with eleven tumors treated, at a median age of 59.5 years (range 46–70 years-old).

A complete otolaryngology examination was performed before RFA treatment to exclude other lesions. A preoperative assessment included location and the tumor size by CT, MR, or ultrasound. For patients with bilateral Warthin tumors, the side with the larger tumor was treated first.

### 2.2. RFA Technique and Anatomic Considerations

Preoperative evaluation included US, CT, or MR imaging to identify the location of the tumor. The epicenters of the tumors in all ten patients were all located in the parotid tail, the retromandibular part of the parotid gland, inferior to the main trunk of the facial nerve. Therefore, RFA was performed from the posterior–inferior site of the mandibular angle to avoid damage to the main trunk of the facial nerve, following MR imaging, for which we used an internally cooled electrode (18 gauge, with 5 mm, 7 mm or 1 cm active tip) with an RF generator (VIVA, STARmed and M2004, RF Medical).

In an outpatient setting, each patient was placed in a supine position with their head tilted to the opposite side. Lidocaine hydrochloride (2%) was subcutaneously injected around the puncture site. In most cases, we firstly aspirated the cystic fluid inside the tumor. Then, under ultrasound guidance, we inserted an electrode parallel to the maximum diameter axis of the tumor, with the tip moving backward and forward as we employed the moving shot technique [15].

Ablation begun with 30 W of RF power. Ablation was considered complete once all tumor compartments showed transient hyperechoic signals on the ultrasound. If a transient hyperechoic zone did not form at the electrode tip within 5–10 s, RF power was increased by 10 W. If the patient reported discomfort, the power was decreased.

All RFA procedures were performed by one of the authors (W.C.L.), who had sixteen years of experience using image-guided procedures.

### 2.3. Postoperative Care

After one hour of close observation, the patients were discharged with ice packing. There were no periprocedural complications. At the beginning of our study, we only administered pain control medications. However, one patient was found to have progressive facial swelling and was thus diagnosed with parotid hematoma 12 days after parotid RFA. After US-guided percutaneous aspiration and antibiotics treatment (Amoxicillin and Clavulanate), that patient recovered in one week. After our experience with that case, prophylaxis antibiotics (Cefadroxil Monohydrate) were given for three days, and a temporary low dose of prednisolone (0.2 mg/kg/day) was given to three of our ten patients due to marked periwound swelling. No other complications, such as facial nerve injury, facial pain, numbness, paresthesia or other minor complications were noted.

### 2.4. Outcome Measurements

Tumor volumes and volume reduction ratios (VRR) were calculated using the following equations. $V=\frac{\pi \alpha \beta \gamma }{6}$, where V is volume, π (pi) is ratio of the circumference of a circle to its diameter, α is the maximum diameter, and β and γ are the other two perpendicular diameters. VRR = (initial volume—final volume)/initial volume × 100%. Regrowth was defined as having a larger volume than the smallest previously recorded volume. All the measurements were performed by two radiologists (W.C.L., Y.C.T.), both with 10–15 years of experience in head and neck imaging.

Warthin tumors have both solid and cystic parts. A cystic component has a high T2 signal intensity on MR imaging and cannot be enhanced on CT or MR. A solid component can be enhanced by CT and MR. A cystic Warthin tumor was defined in this study by presence of a cystic component exceeding 50% of the volume, while a solid Warthin tumor had a cystic component of less than 50%. The retromandibular vein was used to determine the location of the tumor. Tumors located laterally to the retromandibular vein were defined as located in the superficial lobe, whereas others were considered to be located in the deep lobe.

Tumor size, wound condition, cosmetic scale and patient satisfaction were assessed in each patient postoperatively at week one, month one and month six, at least. At enrollment, each patient’s tumor was assigned a cosmetic score: 1 being a non-palpable mass; 2 being an invisible but palpable mass; 3 being a parotid tumor that bulged when teeth were gritted; 4 being a clearly visible mass [14]. Complications were categorized as perioperative (within 1 week), early (after 1 week and within 6 months), or delayed (after 6 months), graded according to The Clavien-Dindo Classification.

### 2.5. Statistical Analysis

For continuous variables, the data were expressed as mean ± standard deviation or median (range, Minimum–Maximum). Pearson’s correlation-coefficient was used to test the correlations between RF parameter and tumor size. Friedman’s one-way analysis was used to compare our longitudinal tumor volume measured by ultrasound at baselines 1 month, 6 months, and ≥12 months. Then, generalized estimating equation (GEE) was used to estimate the trend of volume reduction. The statistical significance of the association between variables and VRR was estimated using an independent sample *t*-test because a normality test had been performed by Kolmogorov–Smirnov test. A two-sided *p* < 0.05 was considered significant. All statistical operations were performed using IBM SPSS Statistics 26.0 (IBM Corp., Armonk, NY, USA).

## 3. Results

RFA operations were performed successfully under local anesthesia on 11 Warthin tumors in ten patients. Patient 10 had two synchronous Warthin tumors on the left side. In patients with bilateral Warthin tumors, the tumors on the other side were small and static, thus requiring no treatment. None of the patients received more than one RFA treatment.

**Figure A1:**
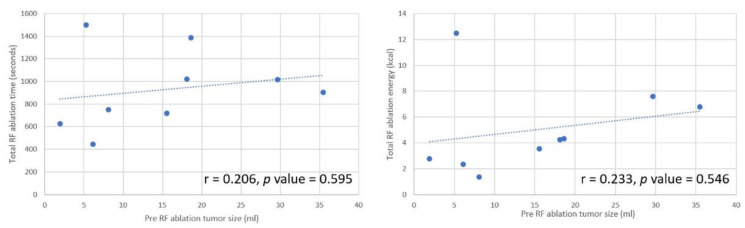
Radiofrequency time and energy had weak positive correlation with tumor size.

As can be seen in Table 1, all patients were males who smoke. Mean follow-up was 24.3 ± 13.1 months (range 7–42). Four of the patients had bilateral Warthin tumors. Four tumors (36%) were located in superficial lobes alone, and seven (64%) in both superficial and deep lobes. Four tumors (36%) were cystic and seven (64%) were solid. Mean ablation time was 15.5 ± 5.8 min and mean total energy deposition was 5.0 ± 3.4 kcal. RF time and energy had a weak positive correlation with tumor size (Pearson Correlation Coefficient = 0.206 and 0.233, *p* = 0.595 and 0.546, respectively, shown in Figure A1). There was no difference in RFA energy, time, and energy delivered per tumor volume between cystic and solid tumors. The early complication rate was 10%, with Patient 6 found to have persistent facial swelling and parotid hematoma twelve days post RFA (Grade IIIa). There were no noted periprocedural or delayed complications.

### 3.1. Efficacy of RFA

**Figure 1:**
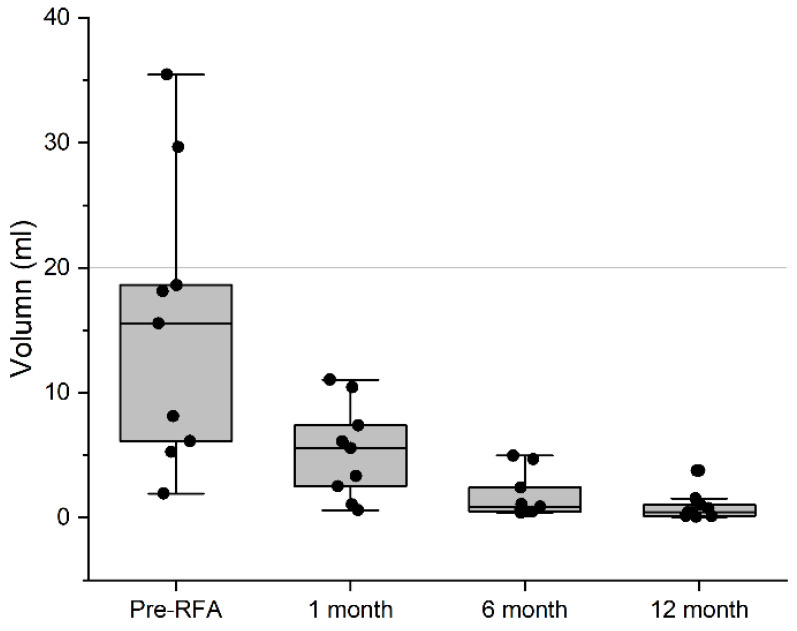
Box plot of Volume Reduction by Time. According to generalized estimating equations, the trend of the decreasing volumes fits a quadratic function (*p* = 0.006), and log transformation has a linear regression (*p* = 0.003).

During the follow-up period (median 22.5 months, range 7–42), no regrowth of residual tumor was found. Mean cosmetic scores, assigned at pre-RFA and postoperative week one, month one and month six, showed marked improvements, with score 4 becoming score 1. Although tumor volume (12.8 ± 11.8 mL) was found to be smaller at the first month (4.3 ± 3.7 mL, *p* = 0.013) and at the sixth month (1.5 ± 1.8 mL, *p* < 0.001), there was no significant difference in volume between the sixth month and ≥12 months (1.0 ± 1.3 mL, *p* = 0.137). Our generalized estimating equations analysis revealed a decreasing trend in volume, fitting a quadratic function (*p* = 0.006). After log transformation, we found the trend to fit a linear function (*p* = 0.003). As can be seen in Figure 1, mean VRR was 86.7% at six months.

### 3.2. Comparison of Outcomes for Tumors That Had Different Consistencies and Locations

**Figure 2:**
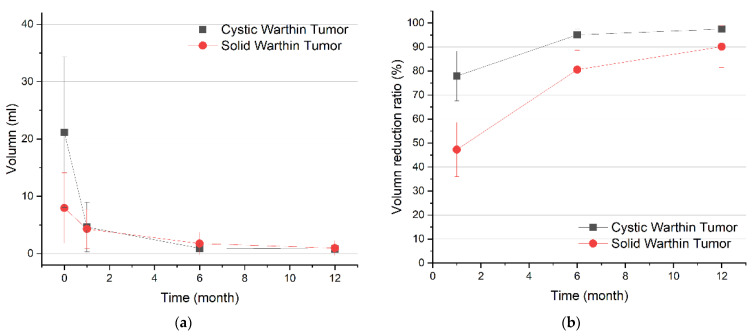
Comparison of outcomes for cystic versus solid Warthin tumors. (**a)** Changes in tumor volume at each follow-up. (**b**) Changes in VRR at each follow-up. VRR = volume reduction ratio.

**Figure 3:**
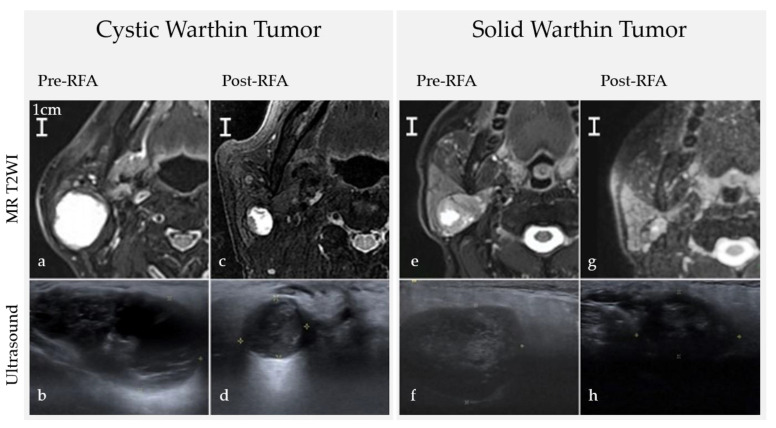
Images before and after RFA comparing consistencies and locations. (**a**,**b**) A huge right cystic Warthin tumor (35.47 mL) (**c**) Post-RFA 8 months VRR = 93.3%. (**d**) Post-RFA 1 months VRR = 68.8%. (**e**,**f**) A right solid Warthin tumor (18.13 mL) (**g**) Post-RFA 12 months VRR = 79.2%. (**h**) Post-RFA 1-month VRR = 51.5%. These two tumors were initially located in both superficial and deep lobes. After RFA, residual tumors were located in superficial lobes only. MR T2WI = Magnetic resonance T2-weighted image.

As can be seen in the summary of the percent changes in Table 2 and Figure 2, and in the image studies of the tumors (Figure 3), cystic tumors had a better VRR than solid tumors at month one and month six follow-ups (cystic vs. solid, 77.9% vs. 47.3%, 95.1% vs. 80.6%, respectively, *p* = 0.003). Tumors located in both deep and superficial lobes were larger than those in superficial lobes alone (Mean volume 18.81 mL vs. 2.18 mL, *p* = 0.014), though no difference in VRR was found. All residual tumors were located in superficial lobes only. Three of the four patients who presented bilateral Warthin tumors at the time of enrollment had new lesions noted on ultrasound during the follow-up period, but no intervention was performed on the new lesions because of their tiny size (less than 0.5 cm in the maximum diameter).

## 4. Discussion

In this study, we updated our previous study of the use of RFA to treat Warthin tumors. Our previous study was of seven patients who were not divided into cystic versus solid groups [14]. In that study, we only followed volume reduction and cosmetic change in patients for six months. In this study, we followed ten patients and divided them into those with cystic and solid tumors for a minimum of seven months, and for some of them, more than 12 months. The results were similar. RFA treatment significantly reduced tumor volume and improved compressive and cosmetic complaints. Local control of Warthin tumors was achieved and maintained over the long-term follow-up (mean 24.3 ± 13.1 months, range 7–42 months).

The effect of RFA seemed to reach a plateau during 6–12 months post operation, as evidenced by the non-significant difference in volume at 6 and 12 months. After log transformation, the GEE model showed a linear regression in residual volume data.

There are still concerns regarding the regrowth of residual tumors remaining after ablation. Reviewing published studies on the treatment of benign thyroid nodules with thermal ablation, we found the recurrence rate to be as high as 5.6% after 1–2 years [16], and regrowth of thyroid nodule started 12 months after RFA, peaking at 2–4 years [17]. As seen in the ultrasonography, most cases of regrowth begin at the tumor margin [18], where the feeding arteries and perinodular veins are located. Unlike benign thyroid nodules, an ablated Warthin tumor has no obvious viable region or vascular structure. In our study, there was no increase in size of RF-ablated Warthin tumors. The recurrence or regrowth of a surgical resected Warthin tumor is rare. Lee et al. (2017) reported 40 cases of extracapsular dissection without any recurrence [19]. Warthin tumors consist mainly of lymphoid tissue and cystic spaces [3], whereas thyroid nodules contain functional glands with high vascularity. Based on our experience, vascular ablation for a Warthin tumor is not necessary. However, a longer follow-up and larger sample size are needed to better assess the risk of regrowth.

Theoretically, during RFA, a slow energy deposition is more effective than a quick temperature rise. At 55 °C, for example, tissue death occurs within two seconds; at 100 °C, tissue death is immediate as evaporation occurs, which is not desired in RFA because of the insulating effect of charred tissue, causing high resistance and difficulty in the transportation of energy to adjacent tissues [20]. In our study, RF time and energy weakly correlated with tumor size. As long as all the targeted tumors elicited hyperechoic signals, the ablation was considered effective and complete, regardless of time and energy. Nevertheless, it is still best to keep the energy to the target region low in order to avoid evaporation of tissue, excessive necrosis, and risk of abscess formation.

Cystic Warthin tumors had better VRR in the first six months after RFA, a result likely related to the cytology structure of the tumor. Macroscopically, Warthin tumors are mainly cystic, usually filled with a brown gelatinous fluid. Regarding histopathology, the tumor has a thin capsule, epithelium, and lymphoid components. The inner structure can be divided into solid and cystic parts. The solid parts are the stroma of lymphoid tissue, where germinal centers are full of mast cells and plasma cells; the cystic spaces contain eosinophilic secretions or amorphous material [3].

Warthin tumors vary widely in their components, such as the proportion of cyst, encapsulation, metaplasia, lymphoid stroma, and epithelium. In the classification by Seifert et al. (1980), a typical Warthin tumor (77%) is defined as having an equal ratio of epithelium to connective tissue [21]. On medical imaging, a cystic Warthin tumor has a loose structure, less cell density, an even conduction of heat, and an absence of a heat sink effect. Therefore, less radiofrequency energy is needed to induce tissue necrosis. This reduction in energy might explain why the cystic tumors in our study had better VRR in the first six months. A similar condition has been noted in RFA treatment of benign thyroid nodules, with mean volume reduction of predominantly cystic thyroid nodules superior to that of solid ones [15,22].

There is some controversy regarding whether a Warthin tumor is actually a true neoplasia, and some studies suggest that it is better viewed as a reactive oncocytic metaplasia accompanied by an inflammation response [23,24]. This theory would better explain the multifocal occurrence of the Warthin tumor, the effectiveness of RFA, and the lack of regrowth after RFA, since there is thermal-injury-induced apoptosis and clearance of inflammatory cells. Thermal-injury-induced apoptosis caused by RFA takes time to resolve, and thus residual tumors continue to decrease in size over time after the procedures.

There were some studies about the cytology and immune response to RFA. For hepatic cellular carcinoma, RFA would induce coagulation necrosis, leading to formation of granulation capsules and infiltration of fibroblasts and mononuclear cells [25]. For the RFA of benign thyroid nodules, the histology features central scattered areas of hyaline sclerosis and scarring [26]. There is no necrosis and formation of the capsule in thyroid nodules after RFA. Therefore, RFA does not affect subsequent thyroid surgery or pathological staging, in case the residual thyroid nodule transforms into a thyroid cancer that needs surgical resection. These above-mentioned histology findings were based on surgical excision of the tumors followed by RFA. As the regrowth rate and risk of malignant transformation was quite low in parotid Warthin tumors, we could not investigate the histopathology of the immune response based on our experience and the medical literature. In post-RFA imaging of this study, there was no fibrosis or necrotic change of the soft tissue surrounding the residual tumors. Further histological studies are needed to confirm the impact of radiofrequency on the microenvironment of Warthin tumors.

The tumors located in both lobes were larger, on average, than those located in the superficial lobes alone. No significant difference was found in VRR between the two groups of tumor locations, probably because there was sufficient ablation of all the components of the tumors. Interestingly, all residual tumors in our study were found in superficial lobes. After RFA, all the tumors shrank and appeared to move towards the superficial lobe, suggesting that RFA is a safe and efficient treatment strategy for tumors in deep lobes of parotid gland. Provided that all residual tumors are located in superficial lobes only, it would be relatively safe and convenient to perform repeated RFA or surgical resections, namely superficial parotidectomy, if needed.

Four of our patients had bilateral Warthin tumors, and three out of these four patients had multifocal lesions, noted on the ultrasonography during regular follow-up. Warthin tumors could be synchronous or metachronous, so repeated treatment might be needed for the new lesions. Patients with multifocal lesions should be actively followed up. In this study, Case 10 presented two separate Warthin tumors on the same side during his first visit, so RFA was performed on both at the same time. Repeated RFA for multifocal Warthin tumors is feasible and superior to traditional surgery because repeated surgery, bilateral parotidectomy or extensive parotidectomy, may increase the risk of cosmetic complaints, Frey’s syndrome, facial palsy, and xerostomia.

In addition to radiofrequency ablation, many kinds of hyperthermia therapy, such as laser ablation, microwave, and focused ultrasound surgery, have been proposed and widely discussed. Different devices and techniques have to be applied to treat different tumors. RFA and percutaneous laser ablation (PLA) are commonly used to treat thyroid nodules, and RFA appears to be slightly superior to PLA, especially for cystic thyroid nodules [27]. Laser interstitial thermal therapy (LITT) was reported to treat recurrent head and neck cancers, minor salivary gland malignancy, and recurrent glioblastomas (GBM) [28,29]. Warthin tumors can alternatively be treated with microwave ablation, with VRR reported to be 53% and 82% at month one and month three, respectively, after the treatment [30]. More research is needed for the utility and comparison between these newly emerging treatment modalities to treat benign tumors and recurrent, unresectable neoplasms.

This study is limited in that it was a retrospective study of a small number of cases with a wide range of follow-up periods, and because it lacked case controls. However, we did show that cystic Warthin tumors had a better volume reduction in the first six months after RFA and that tumors located in both superficial and deep lobes had the same response to RFA as tumors in superficial lobes alone.

## 5. Conclusions

RFA is a safe and effective treatment for Warthin tumors, providing better volume reduction in cystic tumors in first six months. Its therapeutic results are sustainable over the long term, regardless of whether the Warthin tumor is solid or cystic, or located in the superficial lobe alone or in both lobes. RFA is especially useful for multifocal tumors. All residual tumors were found in superficial lobes, making it easier for re-interventions to be carried out if necessary.

## Figures and Tables

**Table 1:** Demographic Information of Patients with Warthin Tumor.

Case	Age (y)/Sex	Comorbidity	Smoke	Image Modality	Bilateral	Tumor Location (Lobe)	Consistency	Follow-Up (Month)	Complication
1	68/M	ESRD	yes	US, CT	-	L supf	Solid	12	Nil
2	70/M	DM, HTN	yes	US, CT, MR	-	R supf	Solid	42	Nil
3	64/M	DM, LC	yes	US, CT, MR	yes	R supf + deep	Cystic	12	Nil
4	46/M	HTN	yes	US, CT	-	R supf + deep	Solid	27	Nil
5	55/M	Nil	yes	US, CT	-	R supf + deep	Solid	41	Nil
6	64/M	Nil	yes	US, CT, MR	yes	R supf + deep	Cystic	36	Yes *
7	54/M	Nil	yes	US, CT, MR	yes	L supf + deep	Solid	33	Nil
8	57/M	DM, HTN	yes	US, MR	yes	R supf + deep	Solid	18	Nil
9	53/M	HTN	yes	US, CT, MR	-	L supf + deep	Cystic	15	Nil
10	62/M	Nil	yes	US, CT, PET	-	L supf	Upper solid	7	Nil
						L supf	Lower cystic		

M = male; DM = diabetes mellitus; ESRD = end-stage renal disease; HTN = hypertension; LC = liver cirrhosis; R = right; L = left; supf = superficial. * The case 6 had RFA at out-patient department and returned to ER due to parotitis with hematoma 12 days after RFA, then treated with ultrasound-guided percutaneous aspiration and antibiotic (Amoxicillin/Clavulanate) for 1 week.

**Table 2:** Comparison of Warthin Tumor by Groups.

		Cystic (*n* = 4)	Solid (*n* = 7)	*p* Value	Supf Lobe (*n* = 4)	Supf + Deep Lobe (*n* = 7)	*p* Value
Median age (range) year		63 (53–64)	57 (46–70)		68 (62–70)	55 (46–64)	
Mean diameter (cm)		4.2 ± 1.8	3.2 ± 1.3	0.286	2.1 ± 0.8	4.4 ± 1.1	0.005 **
Location	Supf lobe alone	1	3		-	-	
	Supf and deep lobe	3	4		-	-	
Consistency	Cystic	-	-		1	3	
	Solid	-	-		3	4	
Mean volume (mL)		21.18 ± 15.18	7.96 ± 6.62	0.071	2.18 ± 2.13	18.81 ± 10.66	0.014 *
Residual volume (mL)	1st month	4.63 ± 5.00	4.31 ± 3.70	0.908	0.94 ± 1.06	6.42 ± 3.60	0.017 *
	6th month	0.95 ± 1.04	1.77 ± 2.11	0.491	0.30 ± 0.23	2.14 ± 1.96	0.042 *
	≥12th months	0.81 ± 0.70	0.98 ± 1.41	0.854	0.29 ± 0.22	1.11 ± 1.28	0.418
VRR (%)	1st month	77.9 ± 12.0	47.3 ± 12.2	0.003 **	57.0 ± 15.0	59.2 ± 22.5	0.871
	6th month	95.1 ± 2.7	80.6 ± 8.8	0.004 **	83.9 ± 8.5	87.0 ± 11.4	0.651
	≥12th months	97.5 ± 1.8	90.1 ± 9.5	0.119	87.4 ± 14.4	94.1 ± 6.9	0.351
Mean Cosmetic score †	Pre-RFA	4	4		4	4	
	Post-RFA	1	1		1	1	
Complication		1	0		0	1	

VRR = volume reduction ratio; supf = superficial. † Cosmetic score: 1, no palpable mass; 2, invisible but palpable mass; 3, bulging appearance of parotid tumor on gritting teeth; 4, obviously visible mass. * *p* < 0.05 (significant), ** *p* < 0.01 (highly significant).

## Data Availability

The data presented in this study are available on request from the corresponding author. The data are not publicly available due to patients’ privacy.

## References

[1] Young A., Okuyemi O.T. (2021). Benign Salivary Gland Tumors. StatPearls.

[2] Sharma M., Saxena S., Agrawal U. (2008). Squamous cell carcinoma arising in unilateral Warthin’s tumor of parotid gland. J. Oral Maxillofac. Pathol..

[3] Barnes L., Pathologie U.-S.Z.D., Eveson J.W., Pathology I.A.O., Sidransky D., Reichart P., World Health Organization (2005). Pathology and Genetics of Head and Neck Tumours.

[4] Maiorano E., Muzio L.L., Favia G., Piattelli A. (2002). Warthin’s tumour: A study of 78 cases with emphasis on bilaterality, multifocality and association with other malignancies. Oral Oncol..

[5] Klussmann J.P., Wittekindt C., Preuss S.F., Al Attab A., Schroeder U., Guntinas-Lichius O. (2006). High risk for bilateral Warthin tumor in heavy smokers—Review of 185 cases. Acta Oto-Laryngol..

[6] Yoo G.H., Eisele D.W., Askin F.B., Driben J.S., Johns M.E. (1994). Warthin’s tumor: A 40-year experience at The Johns Hopkins Hospital. Laryngoscope.

[7] Ruohoalho J., Mäkitie A.A., Aro K., Atula T.S., Haapaniemi A., Keski-Säntti H., Takala A., Bäck L.J. (2017). Complications after surgery for benign parotid gland neoplasms: A prospective cohort study. Head Neck.

[8] O’Brien C.J. (2003). Current management of benign parotid tumors?The role of limited superficial parotidectomy. Head Neck.

[9] Thangarajah T., Reddy V.M., Castellanos-Arango F., Panarese A. (2009). Current controversies in the management of Warthin tumour. Postgrad. Med. J..

[10] Schwalje A.T., Uzelac A., Ryan W.R. (2015). Growth rate characteristics of Warthin’s tumours of the parotid gland. Int. J. Oral Maxillofac. Surg..

[11] Yaranal P.J., Umashankar T. (2013). Squamous Cell Carcinoma Arising in Warthin’s Tumour: A Case Report. J. Clin. Diagn. Res..

[12] Alkan U., Shkedy Y., Mizrachi A., Shpitzer T., Popovtzer A., Bachar G. (2017). Inflammation following invasive procedures for Warthin’s tumour: A retrospective case series. Clin. Otolaryngol..

[13] Bu L., Zhu H., Racila E., Khaja S., Hamlar D., Li F. (2019). Xanthogranulomatous Sialadenitis, an Uncommon Reactive Change is Often Associated with Warthin’s Tumor. Head Neck Pathol..

[14] Tung Y.-C., Luo S.-D., Su Y.-Y., Chen W.-C., Chen H.-L., Cheng K.-L., Lin W.-C. (2019). Evaluation of Outcomes following Radiofrequency Ablation for Treatment of Parotid Tail Warthin Tumors. J. Vasc. Interv. Radiol..

[15] Baek J.H., Kim Y.S., Lee D., Huh J.Y., Lee J.H. (2010). Benign Predominantly Solid Thyroid Nodules: Prospective Study of Efficacy of Sonographically Guided Radiofrequency Ablation Versus Control Condition. Am. J. Roentgenol..

[16] Lim H.K., Lee J.H., Ha E.J., Sung J.Y., Kim J.K., Baek J.H. (2013). Radiofrequency ablation of benign non-functioning thyroid nodules: 4-year follow-up results for 111 patients. Eur. Radiol..

[17] Sim J.S., Baek J.H., Lee J., Cho W., Jung S.I. (2017). Radiofrequency ablation of benign thyroid nodules: Depicting early sign of regrowth by calculating vital volume. Int. J. Hyperth..

[18] Sim J.S., Baek J.H. (2019). Long-Term Outcomes Following Thermal Ablation of Benign Thyroid Nodules as an Alternative to Surgery: The Importance of Controlling Regrowth. Endocrinol. Metab..

[19] Lee D.H., Yoon T.M., Lee J.K., Lim S.C. (2017). Extracapsular dissection for Warthin tumor in the tail of parotid gland. Acta Oto-Laryngol..

[20] Hong K., Georgiades C. (2010). Radiofrequency Ablation: Mechanism of Action and Devices. J. Vasc. Interv. Radiol..

[21] Seifert G., Bull H.G., Donath K. (1980). Histologic subclassification of the cystadenolymphoma of the parotid gland. Virchows Arch..

[22] Jeong W.K., Baek J.H., Rhim H., Kim Y.S., Kwak M.S., Jeong H.J., Lee D. (2008). Radiofrequency ablation of benign thyroid nodules: Safety and imaging follow-up in 236 patients. Eur. Radiol..

[23] Teymoortash A., Schrader C., Shimoda H., Kato S., Werner J. (2007). Evidence of lymphangiogenesis in Warthin’s tumor of the parotid gland. Oral Oncol..

[24] Honda K., Kashima K., Daa T., Yokoyama S., Nakayama I. (2000). Clonal analysis of the epithelial component of Warthin’s tu-mor. Hum. Pathol..

[25] Koda M., Murawaki Y., Hirooka Y., Kitamoto M., Ono M., Sakaeda H., Joko K., Sato S., Tamaki K., Yamasaki T. (2012). Complications of radiofrequency ablation for hepatocellular carcinoma in a multicenter study: An analysis of 16 346 treated nodules in 13 283 patients. Hepatol. Res..

[26] Dobrinja C., Bernardi S., Fabris B., Eramo R., Makovac P., Bazzocchi G., Piscopello L., Barro E., de Manzini N., Bonazza D. (2015). Surgical and Pathological Changes after Radiofrequency Ablation of Thyroid Nodules. Int. J. Endocrinol..

[27] Baek J.H., Lee J.H., Valcavi R., Pacella C.M., Rhim H., Na D.G. (2011). Thermal Ablation for Benign Thyroid Nodules: Radiofre-quency and Laser. Korean J. Radiol..

[28] Montemurro N., Anania Y., Cagnazzo F., Perrini P. (2020). Survival outcomes in patients with recurrent glioblastoma treated with Laser Interstitial Thermal Therapy (LITT): A systematic review. Clin. Neurol. Neurosurg..

[29] Feyh J., Gutmann R., Leunig A., Jäger L., Reiser M., Saxton R., Castro D., Kastenbauer E. (1996). MRI-Guided Laser Interstitial Thermal Therapy (LITT) of Head and Neck Tumors: Progress with a New Method. J. Clin. Laser Med. Surg..

[30] Jin M., Fu J., Lu J., Xu W., Chi H., Wang X., Cong Z. (2019). Ultrasound-guided percutaneous microwave ablation of parotid gland adenolymphoma. Medicine.

